# A multidimensional approach to assessing intervention fidelity in a process evaluation of audit and feedback interventions to reduce unnecessary blood transfusions: a study protocol

**DOI:** 10.1186/s13012-016-0528-x

**Published:** 2016-12-12

**Authors:** Fabiana Lorencatto, Natalie J. Gould, Stephen A. McIntyre, Camilla During, Jon Bird, Rebecca Walwyn, Robert Cicero, Liz Glidewell, Suzanne Hartley, Simon J. Stanworth, Robbie Foy, Jeremy M. Grimshaw, Susan Michie, Jill J. Francis, for the AFFINITIE programme

**Affiliations:** 1 Centre for Health Services Research, School of Health Sciences, City, University of London, London, EC1V 0HB UK; 2 School of Mathematics, Computer Science, Engineering, City, University of London, London, UK; 3 Institute of Health Sciences, University of Leeds, Leeds, UK; 4 National Health Service Blood & Transplant, Oxford Radcliffe Hospitals, University of Oxford, Oxford, UK; 5 Department of Medicine & Ottawa Hospital Research Institute, University of Ottawa, Ottawa, Canada; 6 Centre for Outcomes Research and Effectiveness, University College London, London, UK

**Keywords:** Process evaluation, Fidelity, Audit and feedback, Research protocol, Blood transfusion, Implementation, Randomised controlled trial

## Abstract

**Background:**

In England, NHS Blood and Transplant conducts national audits of transfusion and provides feedback to hospitals to promote evidence-based practice. Audits demonstrate 20% of transfusions fall outside guidelines. The AFFINITIE programme (Development & Evaluation of *A*udit and *F*eedback *IN*terventions to *I*ncrease evidence-based *T*ransfusion pract*I*c*E)* involves two linked, 2×2 factorial, cluster-randomised trials, each evaluating two theoretically-enhanced audit and feedback interventions to reduce unnecessary blood transfusions in UK hospitals. The first intervention concerns the content/format of feedback reports. The second aims to support hospital transfusion staff to plan their response to feedback and includes a web-based toolkit and telephone support. Interpretation of trials is enhanced by comprehensively assessing intervention fidelity. However, reviews demonstrate fidelity evaluations are often limited, typically only assessing whether interventions were delivered as intended. This protocol presents methods for assessing fidelity across five dimensions proposed by the Behaviour Change Consortium fidelity framework, including intervention designer-, provider- and recipient-levels.

**Methods:**

(1) Design: Intervention content will be specified in intervention manuals in terms of component behaviour change techniques (BCTs). Treatment differentiation will be examined by comparing BCTs across intervention/standard practice, noting the proportion of unique/convergent BCTs. (2) Training: draft feedback reports and audio-recorded role-play telephone support scenarios will be content analysed to assess intervention providers’ competence to deliver manual-specified BCTs. (3) Delivery: intervention materials (feedback reports, toolkit) and audio-recorded telephone support session transcripts will be content analysed to assess actual delivery of manual-specified BCTs during the intervention period. (4) Receipt and (5) enactment: questionnaires, semi-structured interviews based on the Theoretical Domains Framework, and objective web-analytics data (report downloads, toolkit usage patterns) will be analysed to assess hospital transfusion staff exposure to, understanding and enactment of the interventions, and to identify contextual barriers/enablers to implementation. Associations between observed fidelity and trial outcomes (% unnecessary transfusions) will be examined using mediation analyses.

**Discussion:**

If the interventions have acceptable fidelity, then results of the AFFINITIE trials can be attributed to effectiveness, or lack of effectiveness, of the interventions. Hence, this comprehensive assessment of fidelity will be used to interpret trial findings. These methods may inform fidelity assessments in future trials.

**Trial registration:**

ISRCTN 15490813. Registered 11/03/2015

**Electronic supplementary material:**

The online version of this article (doi:10.1186/s13012-016-0528-x) contains supplementary material, which is available to authorized users.

## Background

Blood transfusion is one of the most widely and frequently used clinical interventions. However, a substantial proportion of transfusions is considered unnecessary, in that they are administered to patients where clinical studies suggest no clear benefit [1]. Not only are blood components (i.e. red cells, platelets, plasma) scarce and costly resources, but unnecessary transfusions place patients at risk of harm (e.g. of medical adverse events, such as transfusion reactions, or errors in patient identification) [2]. Hence, strategies are needed to reduce unnecessary use of blood components by changing current transfusion practice.

One strategy with the potential to achieve such change is audit and feedback (A&F). A&F is defined as a ‘summary of clinical performance of healthcare over a specified time period, to provide healthcare professionals with data on performance’ [3]. In England, the National Health Service Blood and Transplant (NHSBT) National Comparative Audit (NCA) programme runs national audits designed to assess whether blood components are being used appropriately and safely across clinical specialties [4]. The NCA’s approach to A&F has remained largely unchanged since its establishment in 2003. In brief, an audit-writing group is convened, usually consisting of an audit lead (typically a consultant haematologist with an interest in transfusion), statistician and clinical staff representatives from the clinical specialty being audited (e.g. orthopaedics). This group is responsible for agreeing the audit standards against which clinical practice will be compared, data to be collected, and the findings and recommendations to be included in feedback reports. Resulting feedback reports are subsequently uploaded and delivered to the hospital transfusion team (i.e. transfusion practitioner, consultant haematologist, transfusion laboratory manager) via a hospital-specific NCA audit webpage. It is intended or assumed that this team will subsequently disseminate these reports within their hospitals, and, where feedback indicates discrepancies between current practice and audit standards, lead on a planning process to encourage practice change. However, the NCA currently provides no formal support to facilitate such planning.

Although there have been some incremental improvements (i.e. reductions) in use of blood products over recent years, a consistent finding from these audits is that usage of blood components by different clinical specialities remains higher than indicated by evidence-based guidelines; with approximately 1 in 5 transfusions falling outside national recommendations [5]. This raises questions regarding the effectiveness of current A&F strategies in this context—a concern mirrored in the wider A&F literature [3, 6]. A Cochrane review demonstrated that although A&F interventions have modest, albeit worthwhile, effects on clinical practice (median +4%), outcomes across A&F interventions are highly variable (IQR 0.5 to 16%) [3]. Reasons underpinning this heterogeneity are unclear [3, 7]. It has been argued that the systematic application of evidence and behaviour change theory has the potential to enhance the effectiveness of A&F interventions [6].

### Development & Evaluation of enhanced *A*udit & *F*eedback *I*nterventions to *I*ncrease uptake of evidence-based transfusion pract*i*c*e*: the AFFINITIE programme

**Fig. 1:**
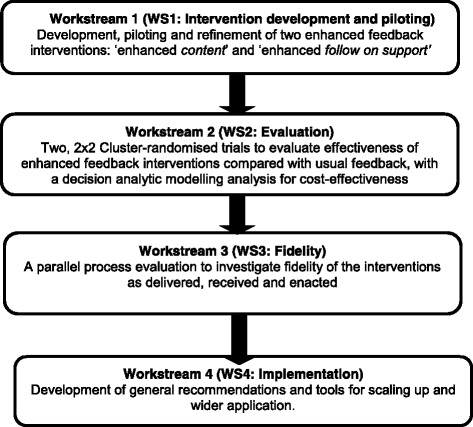
AFFINITIE programme workstreams overview

The AFFINITIE research programme is funded by the UK’s National Institute of Health Research and is conducted in collaboration with NHSBT. AFFINITIE uses the NCA as a platform for applying existing evidence as to what makes feedback more effective (i.e. A&F Cochrane review [3]), and behavioural theory (i.e. control theory [8]) to develop and evaluate two enhanced feedback interventions. AFFINITIE consists of four workstreams (Fig. 1). The protocol for intervention development (Workstream 1; Fig. 1) has been published elsewhere [9].

Briefly, Intervention 1 (‘enhanced content’) concerns the content and format of feedback reports delivered to hospitals. It is a cascade intervention consisting of two components. First, an enhancement guidance manual targeted at the NCA audit-writing group, which includes guidance on how to apply five proposed enhancements for writing feedback reports with evidence- and theory-based content. The proposed enhancements were identified following a content analysis of previous NCA feedback reports. This examined whether effective components of A&F identified in the A&F Cochrane Review, and behaviour change techniques (BCTs) [10] consistent with control theory (e.g. goal setting, feedback, action planning) [7] featured in existing reports. It is intended that the audit-writing group will apply this guidance to produce a template feedback report with enhanced content, which will subsequently be populated with hospital-specific audited data. The second component in the cascade is the resulting feedback report, which will be uploaded to each hospital’s individual NCA webpage, where intervention recipients (i.e. hospital transfusion team) can access and download their reports.

Intervention 2 (‘enhanced follow-on’) concerns the actions taken in hospitals in response to feedback reports and aims to support relevant hospital transfusion staff to plan their response to the feedback. It consists of a web-based toolkit for use by the hospital transfusion team. The toolkit aims to facilitate three behaviours in response to feedback: (i) dissemination of findings to all relevant clinical staff involved in transfusion decision making; (ii) goal setting, problem solving and action planning to facilitate practice changes in response to feedback; and (iii) continued re-monitoring of the clinical practices that were audited. The toolkit will be accessible to hospital staff via a web-link uploaded to the hospital-specific NCA webpage. As a co-intervention to prompt engagement with the toolkit, hospital transfusion teams will receive an initial telephone support call from an intervention provider, offering support and advice on how to use the toolkit. A telephone line will subsequently be available for hospitals to contact intervention providers for further support as needed.

**Fig. 2:**
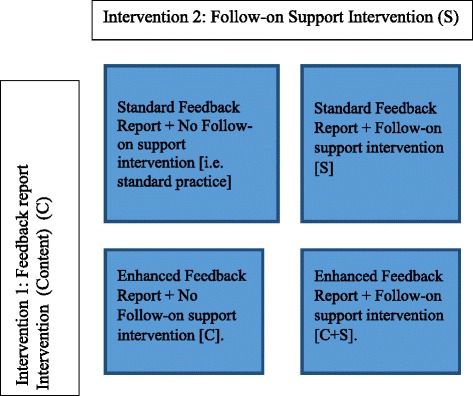
AFFINITIE sequential, replicate 2×2 pragmatic cluster-randomised controlled trial design

The effectiveness and cost-effectiveness of both interventions will be evaluated in two sequential, linked, 2×2 factorial, pragmatic cluster-randomised controlled trials (RCTs) (Workstream 2; Figs. 1 and 2). Trial 1 audits clinical management and transfusion decision-making for elective surgical patients. Trial 2 audits appropriateness of red cell and platelet transfusions in haematology patients. The comparator for both interventions is NCA’s existing, standard A&F practice (i.e. clinically led feedback report; no formalised follow-on support). The primary outcome is the proportion of unnecessary transfusions administered, to be evaluated through a re-audit of key clinical behaviours approximately 9 months following intervention delivery.

However, A&F is a complex, multilevel, multicomponent intervention and is therefore susceptible to variation in implementation. Intervention fidelity refers to ongoing assessment, monitoring and enhancement of the reliability and internal validity of an intervention study [11]. It broadly involves investigating the extent to which interventions are implemented as originally intended [12]. Therefore, the AFFINITIE programme also includes a parallel process evaluation (Workstream 3; Fig. 1) focused on assessing fidelity in each cluster-RCT to explore how the interventions succeed or fail in reducing unnecessary transfusions.

### The AFFINITIE process evaluation

Theoretical models and frameworks of fidelity argue that fidelity is a multidimensional concept, relevant at intervention designer-, provider- and recipient-levels [11]. However, fidelity assessments of complex interventions rarely look at the ‘whole picture,’ and often focus on investigating a single dimension of fidelity; typically whether or not intervention providers delivered interventions as specified, and less so whether intervention recipients comprehend, engage with or enact interventions as intended [13–15]. Assessing recipient-level fidelity is particularly important for interventions such as the AFFINITIE feedback interventions. Both interventions’ components are standardised across recipients and are delivered only once (i.e. both the feedback report link and web link to the toolkit are delivered once, digitally, although both resources are available to recipients throughout the 9-month intervention period). The interventions are thus likely to display limited variability in designer/provider-level fidelity. Rather, it is likely that any variability in observed outcomes may in part be attributable to variation in whether and how transfusion clinical staff receiving the feedback interventions initially understand, engage with and subsequently enact the interventions in day-to-day clinical practice [16].

Therefore, we aim to adopt a multidimensional framework to assess fidelity, by applying the US National Institute of Health Behaviour Change Consortium’s (BCC) fidelity framework and guidance [11]. This framework proposes five fidelity dimensions: (1) *Design:* treatment differentiation, acceptability, comprehensive specification of intervention components, theoretical underpinning and causal assumptions; (2) *Training:* extent to which intervention providers are competent and adequately trained to deliver interventions; (3) *Delivery:* extent to which intervention content is delivered as intended during the intervention period; (4) *Receipt*: whether intervention recipients initially understand/ engage with the intervention; and (5) *Enactment:* extent to which recipients apply the intervention as intended in ‘real life’ or target settings (e.g. clinical practice). It is important to assess fidelity across all five dimensions, as lack of fidelity to just one could affect study outcomes and thus the internal validity of the trial [12].

Moreover, outcomes and enactment across participants may be facilitated or hindered by contextual factors external to the intervention (e.g. organisational norms, policy, available resources) [17]. In AFFINITIE, external factors, such as the publication of new transfusion clinical guidelines [18], may impact on responses to the interventions and observed outcomes. Some factors may influence intervention and control arms equally, while others may interact with or duplicate active components in the interventions. Therefore, to facilitate interpretation of fidelity and outcome data, a secondary objective of the AFFINITIE process evaluation is to explore potential contextual factors influencing fidelity of receipt and enactment.

### Aims

The aims of the study are to conduct a multidimensional assessment of fidelity for two enhanced feedback interventions and assess the extent to which fidelity contributes to observed outcomes (i.e. proportion of unnecessary transfusions). The specific research questions map onto the BCC fidelity dimensions:

1. To what extent: (i) are the two enhanced feedback interventions clearly specified a priori, and (ii) is it possible to differentiate between the components of the two enhanced interventions and standard practice comparators (*design*)?
2. To what extent have the feedback interventions been delivered as intended by intervention providers (*training/delivery)*?
3. To what extent were the feedback interventions received and enacted as intended by transfusion clinical staff in UK hospitals (*receipt/enactment)*?
4. What are the contextual influences that might have influenced clinical staffs’ responses to the feedback interventions?
5. To what extent do measures of fidelity mediate observed outcomes?

## Methods

### Overall process evaluation design

Two linked, mixed-method fidelity assessments will be conducted in parallel to the two linked cluster-RCTs. Table 1 outlines how each sub-dimension of the five BCC fidelity dimensions [11] will be addressed in AFFINITIE. A summary of how each fidelity dimension will be investigated will be presented in turn.

**Table 1:** BCC fidelity dimensions (Bellg et al. [11]) and their application in the AFFINITIE trial

Fidelity dimensions	Application intervention 1 (enhanced content—feedback reports)	Application intervention 2 (enhanced follow-on support—web-based toolkit + telephone support)
Design		
Provide information about treatment dose in intervention and control/comparison condition: length of contacts (min), number of contacts, content of treatment, duration of contact over time	• Intervention content and delivery parameters described in separate intervention development papers for each intervention • Description of intervention content in terms of component behaviour change techniques (BCTs) using established BCT taxonomy • Treatment differentiation: comparison of BCTs between both interventions and between each intervention and corresponding current standard practice comparator. BCTs will be compared in terms of frequency, mode of delivery, behavioural specificity and enactment instruction. BCTs that are identified at least once in either intervention and/or comparator will be classified as either fully convergent (present in similar frequency/mode of delivery/behavioural specificity/enactment instruction in both intervention/comparator), partially convergent (present in both, but at different frequencies/ modes of delivery/ behavioural specificity/ enactment instruction) or unique (present in only intervention or comparator). The percentage of BCTs in each category will be assessed, with a higher proportion of fully/partially convergent BCTs indicating lower treatment differentiation.	
Method to ensure dose is equivalent between conditions.	• Hospitals in the intervention and control trial arms for intervention 1 will both receive at least one feedback report and feedback PowerPoint presentations as per standard practice. • However, dose may differ in terms of the number of feedback reports received per condition, as the enhanced feedback report condition includes multiple feedback reports following a graded entry approach (i.e. level 1—key findings → level 3—detailed supplementary findings report).	• N/A; All hospitals randomised to the enhanced condition for intervention 2 will receive the toolkit and initial telephone support at equivalent doses. However, hospitals in the control condition for intervention 2 will not receive an equivalent dose of intervention 2 as the comparator is a standard practice/no intervention condition.
Method to ensure dose is equivalent for participants within conditions.	• The enhancement guidance manual will be used to produce a report template containing the proposed enhancements, which will be populated with hospital specific data for each hospital. Using a template report will help ensure the format and content of reports is consistent across hospital specific reports.	• The same web-based toolkit will be delivered to all intervention 2 hospitals. Dose is standard within condition. • All hospitals will receive one initial facilitator-initiated telephone support call.
Specification of intervention provider credentials that are needed	• Described under *training* dimension	
Theoretical model upon which the intervention is based is clearly articulated: - The active ingredients are specified and incorporated into the intervention. - Use of experts or protocol review group to determine whether the intervention protocol reflects the underlying theoretical model or clinical guidelines.	• Intervention causal assumptions, theory (control theory) and evidence base (Cochrane audit and feedback review) summarised in logic models reported in intervention development papers. • Component BCTs in each intervention mapped onto control theory • Interventions developed in collaboration with multidisciplinary consensus panel (transfusion clinical staff, behavioural scientists, patient representatives) to ensure the interventions reflect the underlying theoretical models and hold clinical face validity	
Potential confounders that limit the ability to make conclusions at the end of the trial are identified.	• Possible contamination threats (e.g. regional transfusion committee meetings) will be continuously monitored and documented throughout the AFFINITIE trials. • Wider contextual factors external to the AFFINITIE trials that may influence intervention outcomes will be examined via the process evaluation (e.g. publication of new NICE transfusion clinical guidelines).	
Plan to address possible setbacks in implementation (i.e. back-up systems or providers)	• NHS Blood and Transplant and the National Comparative Audit have employed extra staff to support the conduct of the audits and trial data collection. IT staff are available to support systems issues for report upload. These measures will help ensure the audits keep to timeline.	• NHS Blood and Transplant and the National Comparative Audit have employed extra staff to support the conduct of the audits and trial data collection. These measures will help ensure the audits keep to timeline. IT staff have also been appointed to support the maintenance of the web-based toolkit. • Further telephone support will be available, whereby hospitals may speak to an intervention provider to discuss any issues encountered with using the web-based toolkit.
If more than one intervention is described, all interventions are described equally well	• Intervention will be described in intervention development paper using relevant reporting checklist (identified via the Enhancing the Quality and Transparency Of Health Research network http://www.equator-network.org/home/)	• Intervention will be described in intervention development paper using relevant reporting checklist (identified via the Enhancing the Quality and Transparency Of Health Research network http://www.equator-network.org/home/)
Training		
Description of how providers will be trained (manual of training procedures)	• Training procedures outlined in intervention development papers • Training log to document training delivered for each intervention	
Standardisation of provider training (especially if multiple waves of training are needed for multiple groups of providers)	• Standardised training materials, including an enhancement guidance manual and prototype training reports • One full-day training workshop delivered by intervention developers for enhanced audit writing group intervention developers	• Standardised training materials, including a telephone support manual and flow chart • Three training sessions delivered by intervention developers to intervention facilitators
Assessment of provider skill acquisition	• Preliminary draft enhanced reports will be content analysed to assess the extent to which proposed enhancements and theoretically consistent BCTs have been applied as intended • Documented in a skill acquisition record form	• Intervention facilitators role-play delivery of telephone support sessions to a range of possible scenarios • Assess extent to which intended BCTs are delivered according to the telephone support manual • Documented in skill acquisition record form
Characteristics being sought and/or avoided in a treatment provider are articulated a priori.	• Not met: audit lead and report writing group recruited by the National Comparative Audit of blood transfusion as per standard audit practice	• Requisite knowledge and skills for intervention providers outlined in job description
At the hiring stage, assessment of whether or not there is a good fit between the provider and the intervention (e.g. ensure that providers find the intervention acceptable, credible and potentially efficacious).	• Not met: audit lead and report writing group recruited by the National Comparative Audit of blood transfusion as per standard audit practice	• Interview questions and tasks (e.g. BCT coding exercise) were chosen to assess whether applicants for the intervention facilitator roles possessed requisite knowledge and skills in behaviour change.
Delivery		
Method to ensure that content and dose of intervention is delivered as specified.	• Provision of an enhancement guidance manual and enhancement application ‘checklist’	• Telephone support manual, telephone support flow chart and BCT checklist • Telephone support delivery log
Assessment and monitoring of provider skill maintenance over time	• Promoted by intervention developers providing ongoing support to audit writing group during preparation of the reports (i.e. iterative review of draft reports and feedback on how to increase delivery of proposed enhancements and theoretically consistent BCTs)	• Analysis of sub-sample of audio-recorded telephone support sessions • Assess the extent to which BCTs according to the telephone support manual and flowchart are delivered across intervention delivery period
Mechanism to assess if the provider actually adhered to the intervention plan/use of treatment manual/whether active ingredients delivered	• Content analysis of template for enhanced reports to check for application of intended enhancements	• Content analysis of toolkit + sub-sample of audio-recorded telephone support sessions to check that intended BCTs delivered
In the case of computer delivered interventions, method to assess participants’ contact with the information	• See receipt component; web-analytics data will be collected on participant engagement with interventions in terms of number of downloads of each feedback report from the hospital web-page	• See receipt component; web-analytics data will be collected on participant engagement with interventions in terms of number of log ins into the toolkit
There is a plan for the assessment of whether or not proscribed components were delivered (e.g. components that are unnecessary or unhelpful)	• The enhancement guidance specifies three different graded entry levels of feedback reports and outlines the format and BCTs that are to be delivered in each report. A content analysis will be conducted of the template for the different levels of the feedback reports to verify whether any proscribed components were included in each report • Content analysis of feedback reports delivered to standard/comparator arm to monitor for possible contamination/loss of treatment differentiation in terms of BCTs delivered	• Telephone support providers have been instructed to maintain treatment discrimination between the trial arms by not discussing what is being delivered in other trial arms when providing telephone support. A sub-sample of telephone support sessions will be audio-recorded and examined for disclosure of content delivered across trial arms
There is a plan for how contamination between conditions will be prevented	• Two separate but equivalent feedback report writing groups have been established to write the enhanced and standard feedback reports • A protocol for documenting and monitoring possible sources of contamination has been developed and will be implemented throughout the trial. AFFINITIE does not aim to prevent contamination, but rather document it	• Intervention facilitators will deliver telephone support on two separate proactive and reactive telephone support lines (one for hospitals receiving enhanced reports, and one for hospitals receiving standard reports) • A protocol for documenting and monitoring possible sources of contamination has been developed and will be implemented throughout the trial
There is an a priori specification of treatment fidelity (e.g. providers adhere to delivering >80% components)	• Using general consensus criteria, 80–100% adherence to intervention components will be classed as high fidelity; 51–79% moderate; and <50% low fidelity	
Method to ensure that content and dose of intervention is delivered as specified	• Content analysis of template for enhanced reports to check for application of intended enhancements • Examination of each NCA hospital web-page to check whether the intended feedback report(s) were uploaded according to trial arm allocation	• Content analysis of toolkit + sub-sample of audio-recorded telephone support sessions to check that intended BCTs delivered • Examination of each NCA hospital web-page to check that a link to the web-based toolkit was delivered to the intended hospitals according to trial arm allocation •Telephone support delivery log
Receipt		
There is an assessment of the degree to which participants understood the intervention	• Questionnaires sent to clinical staff in all participating hospitals three months post intervention delivery to assess the extent to which they understood the key findings and recommendations in the feedback reports • Semi-structured interviews with clinical staff in a sub-sample of hospitals to examine in greater depth the extent to which clinical staff understood the content of the feedback reports	• Questionnaires sent to clinical staff in all participating hospitals allocated to receive intervention 2 3 months post intervention delivery to assess the extent to which they understood how to use the toolkit • Semi-structured interviews with clinical staff in a sub-sample of hospitals to examine in greater depth the extent to which the clinical staff understood how to use the toolkit
There is specification of strategies that will be used to improve participant comprehension of the intervention	• The enhanced feedback reports were piloted in four hospitals to assess feasibility and acceptability. The reports and enhancement guidance were revised post piloting accordingly, to maximise comprehension and acceptability	• The toolkit was piloted in four hospitals to assess feasibility and acceptability. The toolkit was revised post piloting accordingly, to maximise comprehension and acceptability • Subsequent versions of the toolkit were subject to additional usability testing in hospitals and refined as necessary prior to trial
The participants’ ability to perform the intervention skills will be assessed during the intervention period	• Intervention 1 is delivered only once, and there are no pre-specified skills hospitals are expected to perform for Intervention 1 during intervention delivery.	• Web analytics of toolkit data (e.g. extent of completion of the dissemination, goal setting, problem solving and action planning tasks)
A strategy will be used to improve subject performance of intervention skills during the intervention period.	• Not met: intervention 1 is only delivered once; no additional contacts will be made with hospitals by the audit writing group following delivery of the feedback reports	• Intervention 2 as a whole aims to improve transfusion clinical staffs’ response to feedback by supporting clinical staff to plan their dissemination and response to feedback. • Provision of an initial facilitator-initated telephone support session to all hospitals allocated to receive intervention 2, to encourage engagement with the toolkit and discuss any initial issues or questions • Additional telephone support will be available on an as needed basis to all hospitals receiving intervention 2 for 3 months following intervention delivery
Multicultural factors considered in the development of and delivery of the intervention (e.g. provide in native language; protocol is consistent with the values of the target group)	• The enhanced A&F interventions were developed in continuous consultation and discussion with the range of clinical staff involved in transfusion (i.e. consultant haematologists, lab managers, transfusion practitioners, nurses, etc.). • Interventions were piloted to assess feasibility and acceptability in this clinical context and refined as necessary to address any emerging issues.	
Enactment		
Strategy to assess intervention recipients’ performance of the intervention skills in settings in which intervention might be applied.	• Questionnaires are sent to clinical staff in all participating hospitals 3-month post intervention delivery to assess the extent to which they read the feedback reports, discuss the feedback reports with colleagues and implement any change in light of the findings/ recommendations provided and possible contextual influences on enactment. • Semi-structured interviews with clinical staff in a sub-sample of hospitals to examine in greater depth the extent to which they read the feedback reports, discussed the feedback reports with colleagues and implemented any change in light of the findings/recommendations provided.	• Questionnaires are sent to clinical staff in all participating hospitals allocated to receive intervention 2 3-month post intervention delivery to assess the extent to which they disseminated the feedback, implement any developed action plans, re-monitor their clinical practice using the tools provided and possible contextual influences on enactment • Semi-structured interviews with clinical staff in a sub-sample of hospitals to examine in greater depth the extent to which they disseminated the feedback, implemented any developed action plans and re-monitored their clinical practice using the tools provided

### Fidelity of design

Considerations regarding fidelity of design fall broadly into two categories: (1) *trial* design and (2) *intervention* design [11]. The internal validity of any study will be impacted by whether the selected trial design can adequately test proposed hypotheses. In AFFINITIE, this has been considered as part of Workstream 2 (Fig. 1), via the selection of a 2×2, factorial, cluster-RCT design, and will be discussed in a separate trial protocol.

Fidelity of intervention design concerns how comprehensively interventions are specified a priori, whether intervention components adequately reflect underlying theory, and intervention acceptability [13]. Such considerations have been addressed as part of intervention development in Workstream 1 (Fig. 1). Intervention delivery parameters (i.e. dose/ duration/ number of contacts) and content (i.e. component BCTs) have been specified a priori using BCT labels and definitions from an established BCT taxonomy [10]. The BCTs in each intervention were then mapped onto control theory [8]. Throughout the intervention development process, multidisciplinary experts (behaviour change scientists, transfusion clinical staff, patient representatives) have iteratively reviewed the interventions to consider whether the interventions have clinical face validity and reflect their proposed theory- and evidence-base [3, 8]. Pilot work has been conducted to assess the feasibility and acceptability of the interventions using think-aloud protocols [19] and semi-structured interviews [9].

An additional fidelity of design consideration is treatment differentiation, defined as the degree to which treatments (i.e. interventions), or two or more trial arms differ as intended along critical dimensions (e.g. content) [12]. Treatment differentiation is of particular importance to the AFFINITIE pragmatic cluster-RCTs, which aim to evaluate the effectiveness of two feedback interventions (individually and combined), against current standard NCA feedback practice (Fig. 2). The ability to meaningfully conduct such evaluations depends on a minimum degree of differentiation between the interventions and standard practice comparators.

Treatment differentiation of the interventions as designed will be assessed by conducting comparisons between the content of (1) intervention 1 ‘enhanced content’ vs current standard practice (i.e. clinically led feedback reports); (2) intervention 2 ‘enhanced follow-on support’ vs current standard practice (i.e. no formalised follow-on support); and (3) intervention 1 ‘enhanced content’ vs intervention 2 ‘enhanced follow-on support.’ The intended content of each intervention and comparator will be coded by two independent coders into component BCTs using a BCT taxonomy [10]. BCTs will be compared across both interventions and standard practice comparators in terms of BCT frequency, mode of delivery, behavioural specificity—including relation to target behaviours and enactment instructions. BCTs that are identified at least once in either intervention or comparator will be categorised as ‘fully convergent’ (i.e. present in both the intervention and comparator with similar frequency, mode of delivery, extent of behavioural specificity and enactment instructions); ‘partially convergent’ (i.e. present in both the intervention and comparator, but with differing frequencies, modes of delivery, extent of behavioural specificity and enactment instructions), or ‘unique’ (i.e. present only in the intervention or comparator). For each of the three comparisons, the percentage of BCTs in each of these categories will be noted. A higher proportion of ‘fully’ or ‘partially convergent’ BCTs indicates lower treatment differentiation. Inter-rater coding reliability will be assessed using Cohen’s kappa, with a value of *k* = 0.75 taken to represent high agreement [20].

### Fidelity of training

The BCC guidance advocates standardisation of training between intervention providers and checking providers acquire pre-specified competence indicators prior to intervention delivery [11].

The intervention providers for intervention 1 are the NCA-assembled audit-writing groups. There will be two writing groups: an enhanced writing group, tasked with drafting feedback reports using the enhancement guidance manual; and a standard writing group, tasked with drafting feedback reports as per usual NCA practice. The enhanced writing group will be trained via a full-day workshop, where intervention developers will deliver a presentation explaining each proposed enhancement, and facilitate wider discussion of prototype enhanced feedback reports and how these may be adapted to write feedback reports for the current audit topic. Initial skill acquisition will be assessed by conducting a content analysis of the first draft enhanced feedback report(s). Two independent raters will assess whether the five proposed enhancements and theoretically consistent BCTs are ‘present’, ‘absent but should be present’, or ‘not applicable’ in the draft report, and inter-rater agreement assessed using Cohen’s kappa. The percentage of applicable intervention components present will be calculated.

The intervention providers for intervention 2 are responsible for developing the web-based toolkit (i.e. health psychologists, web designers) and providing the telephone support co-intervention (i.e. health psychologists). Intervention providers will be trained to deliver telephone support by an intervention developer over three training sessions using a standardised telephone support manual and practice role plays. This manual specifies the intended content and format of telephone support calls in terms of component BCTs. Intervention providers’ skill acquisition will be assessed prior to intervention delivery via audio-recorded, simulated role plays of telephone support sessions. An independent rater will content analyse role play transcripts and score the proportion of manual-specified BCTs delivered as intended.

### Fidelity of delivery

Assessing fidelity of delivery during the intervention period concerns whether interventions were delivered as intended (i.e. adherence), ongoing treatment competency (i.e. whether providers maintain skills acquired during training) and monitoring for potential loss of treatment differentiation between trial arms, due to delivery of additional, unspecified intervention components and/or potential contamination [11–13].

For intervention 1, in the intervention arm, ongoing treatment competency will be encouraged by intervention developers providing on-going support to the audit-writing group during the drafting of feedback reports. Intervention developers will iteratively review drafts of reports and provide feedback on how to further incorporate delivery of proposed enhancements and theoretically consistent BCTs. Subsequent fidelity of delivery (i.e. adherence) will be assessed by conducting a content analysis of the final template enhanced feedback report(s). The template report(s) will be coded by two independent raters using a BCT taxonomy [10] to verify whether the five manual-specified enhancements and theoretically consistent BCTs are either ‘present’, ‘absent but should be present’ or ‘not applicable’. Inter-rater reliability will be assessed using Cohen’s kappa. Any additional, non-manual specified BCTs present in the final template feedback report(s) will be documented. The template feedback reports for the standard/comparator arm will be equivalently content analysed to monitor for any contamination and loss of treatment differentiation. To monitor delivery of feedback reports by NHSBT’s NCA, the webpage for every hospital in each trial arm will be examined to verify that the correct feedback reports were uploaded according to cluster allocation (i.e. enhanced vs standard).

For intervention 2, fidelity of delivery for the final version of the toolkit will be examined by conducting a content analysis of the toolkit to assess whether intended and theoretically consistent BCTs are present, absent but should be present or not applicable. The NCA webpage for all participating hospitals will be examined to verify that a link to the toolkit is present for hospitals allocated to receive intervention 2 and absent for control hospitals. For telephone support, fidelity of delivery will be promoted through use of a manual and flow chart to prompt delivery of intended BCTs. A log of all delivered telephone support calls will be maintained and reviewed to monitor whether all participating hospitals allocated to receive intervention 2 received at least one initial telephone support call. All telephone support sessions will be audio-recorded. Adherence and ongoing treatment competency will be assessed by transcribing a randomly selected sub-sample of sessions (*n* = 18 in total; i.e. 9 per trial arm receiving intervention 2; 6 per intervention provider). Transcripts will be coded into component BCTs to monitor whether intervention providers continue to competently deliver BCTs according to manual specification. Both content analyses of the toolkit and telephone support transcripts will be conducted by two independent raters and inter-rater reliability assessed using Cohen’s kappa.

Fidelity of delivery will be quantified for both interventions by assessing the proportion of manual-specified content (i.e. % proposed enhancements/BCTs) delivered as intended in the feedback reports/toolkit/telephone support sessions, and also the proportion of hospitals that received feedback reports, links to the toolkit and/or telephone support, according to trial allocation. Consensus in the literature suggests that 80 to 100% adherence to intervention specifications represents ‘high’ fidelity of delivery, 51 to 79% represents ‘moderate’ fidelity, and <50% or less represents ‘low’ fidelity [12, 21].

### Fidelity of receipt, enactment and contextual influences

Receipt and enactment will be concurrently investigated alongside contextual influences. Recommended strategies for assessing receipt and enactment include objective verification of intervention implementation where possible or recipient self-report [11–13]. Receipt, enactment and context will thus be assessed using a mixed methods approach, combining objective web-analytics data, questionnaires and semi-structured interviews.

#### Web-analytics data to assess engagement and enactment

For intervention 1, the NCA webpage has been programmed to record the number of times each feedback report was downloaded by each hospital throughout the intervention period. For intervention 2, the toolkit has been programmed to record usage patterns during the intervention period, including the number and duration of logins, page views, and indicators of completion of tools (i.e. adding/deleting characters). Patterns of engagement throughout the intervention period will be examined for both interventions and represented visually using graphs.

#### Questionnaires and semi-structured interviews

Questionnaires will assess receipt, enactment and contextual influences across all participating hospitals. Approximately 3 months following intervention delivery, an invitation to complete a web-based questionnaire will be emailed to the NCA-listed clinical contact for the audit at each participating site (i.e. nominated member of the hospital transfusion team, typically transfusion practitioner, leading audit data collection and response). To also collect responses from the range of clinical staff potentially involved in applying findings from the audit, the initial contact will be asked to forward the questionnaire link and invitation email to at least two other clinical staffs involved in transfusion—ideally a further member of the hospital transfusion team and one representative from the clinical specialty being audited (e.g. anaesthetists/ surgeons for trial 1, which audits surgical patient blood management). Respondent demographic characteristics, including role/clinical specialty, will also be recorded. The response rate will be calculated as the percentage of participating sites (*n* = 155 Trial 1; *n* = 167 trial 2) for which at least one questionnaire response is received.

Semi-structured interviews will be conducted in a sub-sample of hospitals to enable more in-depth exploration of receipt, enactment and contextual influences. Interviews lasting up to 1 h will be conducted via telephone approximately 3 months following intervention delivery, with up to three clinical staff members in three randomly selected hospitals per trial arm (i.e. 3 hospitals × 4 trial arms = 12 hospitals; 12 hospitals × 3 clinical staff = 36 participants; 24 hospitals and 72 participants across both trials). Clinical staff eligible to participate will include two members of the hospital transfusion team and a clinical staff member from the relevant specialty/topic being audited.

Questionnaire items and interview topic guides will be based on the Theoretical Domains Framework (TDF) [22, 23] to enable systematic identification of barriers/enablers to intervention recipients understanding, engaging with and/or enacting feedback interventions as intended. The TDF synthesises 128 constructs from 33 distinct behaviour change theories into 12 theoretical domains. The TDF has been applied to investigate barriers/enablers to behaviour change across a range of clinical contexts, including transfusion [24–26]. It has also been applied as part of intervention development in AFFINITIE (Workstream 1; Fig. 1) to identify barriers/enablers to how hospitals currently respond to transfusion A&F delivered by NHSBT’s NCA [9]. The target behaviours of interest are the extent to which clinical staff involved in transfusion decision making for elective surgical (trial 1) or haematology patients (trial 2), initially understand and engage with the feedback materials delivered by the NCA and subsequently act on recommendations provided in the reports. Questionnaire and interview topic guides will include at least one question related to each of the 12 theoretical domains. Additional file 1 provides sample questions for each domain.

Items related to fidelity of receipt will explore the extent to which participants initially engaged with and understood the intervention. TDF domains of relevance to receipt might thus include ‘knowledge’ (e.g.what the key findings and recommendations from the feedback reports were; whether participants understood how to use the tools in the toolkit), and ‘memory, attention, decision making’ (e.g. how readily participants were able to extract key information for their hospital from the feedback materials). Items related to enactment will explore how participants have subsequently acted upon the feedback. TDF domains of relevance to enactment might thus include ‘motivation and goals’ and ‘behavioural regulation’ (e.g. whether they have set goals or action plans in light of feedback; If not, why not? Have they encountered any difficulties when trying to change practice in light of feedback? If so, how have they overcome these?), or ‘nature of the behaviour’ (e.g. how has your response to this audit differed from what you normally do when receiving blood transfusion feedback from the NCA?). The TDF will also allow for potential contextual factors influencing intervention enactment to be investigated, for instance, via the domains ‘social Influences’ (e.g. How receptive have your clinical colleagues been to making changes in response to the audit findings?) and ‘environmental context and resources’ (e.g. To what extent does your team/site have the necessary resources to change practice in light of feedback? If not, what additional resources would be required?) The questionnaires and topic guides will be piloted and reviewed by two haematologists to ensure clinical face validity.

Questionnaire responses and web-analytics data will be summarised descriptively. Transcripts of audio-recorded interviews will be analysed using content analysis following a framework analysis approach [27, 28]. Using the TDF as a coding framework, participant responses within each transcript will be coded into TDF domains they are judged to represent. For example, ‘I do not feel confident that I will be able to act on any of the recommendations provided in the feedback reports’, would be coded as TDF domain ‘beliefs about capabilities’. A 20% sub-sample of transcripts will be double-coded to assess inter-rater reliability using Cohen’s kappa. Responses coded into each domain will subsequently compared across transcripts, sorting responses expressing similar views into groups. A belief statement will be inductively generated for each cluster of grouped responses, summarising the shared view across participants as to the role that domain plays in hindering or enabling intervention enactment. Generated belief statements will then be reviewed to identify those belief statements representing the likely most important barriers/enablers based on three criteria: (1) belief statement frequency across participants; (2) presence of conflicting beliefs across participants; and (3) expressed importance by participants [27].

### Association with outcomes

Systematic reviews demonstrate that few fidelity assessments examine associations between fidelity and outcomes [29]. After quantitative process evaluation data (i.e. extent of fidelity of delivery, receipt/enactment questionnaire responses; web-analytics data) and outcome data (i.e. proportion of unnecessary transfusions) have been analysed, we will explore whether fidelity mediates the impact of the randomised interventions on trial outcomes using causal mediation analyses [30].Full details will be included in the statistical analysis plan for the trials.

### Triangulation

In line with recommendations for mixed methods research, qualitative (e.g. semi-structured interviews) and quantitative (e.g. questionnaires, web-analytics) findings will be integrated following a triangulation protocol (i.e. methodological triangulation) [31–33]. To examine consistencies in key fidelity findings across the two audit topics being evaluated, findings will also be triangulated across trials 1 and 2 (i.e. data source triangulation) [32].For each key finding, data from each data set will be tabulated in a ‘convergence coding matrix’. The extent of similarity of findings across data sets will be compared and categorised as representing either full agreement (data convergence), partial agreement (complementarity between data), conflicting findings (discord) or silence (finding identified in only one data source and no additional sources) [31, 32, 34]. Triangulated findings will be presented schematically to illustrate the relationship between fidelity dimensions and outcomes.

### Trial status

At the time of submission of this protocol (November 2016), the two enhanced A&F interventions had been delivered for both trials. Fidelity data on training, delivery, receipt and enactment had been collected for trial 1. Only data on fidelity of training and delivery had been collected for trial 2. Outcome data collection had commenced for trial 1 but not trial 2. No outcome or fidelity data from either trial were cleaned or analysed prior to submission.

## Discussion

This protocol describes planned strategies for assessing intervention fidelity as part of the process evaluation of two linked cluster RCTs in the AFFINITIE programme. A strength of the present process evaluation is the multidimensional approach to fidelity data collection. The planned methods and five fidelity dimensions assessed are also in line with the four process evaluation functions proposed by the recently published Medical Research Council guidance for designing and conducting process evaluations of complex interventions [35]: (1) ensuring interventions’ core components, theoretical underpinning and key assumptions are clearly articulated (i.e. Design); (2) examining extent of implementation during the intervention period (i.e. training/delivery); (3) examining contextual factors affecting intervention delivery and functioning; and (4) investigating mechanisms of impact through which observed outcomes are achieved, including extent of engagement by intervention recipients (i.e. receipt/enactment; mediation analyses).

Results from the fidelity assessment will contribute to an in depth investigation of *how* the interventions ‘worked’ (or not), which will contribute to interpretation of observed trial outcomes. There are a number of possible scenarios:

1. If the trial outcome identifies significant reductions in the proportion of unnecessary transfusions, and the proposed fidelity assessments identify the interventions were implemented with high fidelity, with minimum contextual barriers, and that extent of fidelity mediated observed outcomes, then it may be more confidently inferred that observed outcomes are attributable to the enhanced A&F interventions.
2. Alternatively, if the interventions are not effective and there is evidence that fidelity was poor or inconsistent, it would be premature to dismiss the interventions as ineffective; given they were not implemented as intended and thus not evaluated. It may also be the case that poor fidelity is associated with delivery or enactment of unplanned BCTs that may have negatively impacted outcomes.
3. Conversely, the trial may identify significant reductions in unnecessary transfusions, despite poor fidelity. If so, delivery of unplanned BCTs or contextual factors may have interacted with intervention delivery to influence practice (e.g. transfusion clinical staff in intervention hospitals being more motivated to change than those in the control hospitals as a result of more contact with intervention providers). Alternatively, the interventions may have operated through mechanisms and pathways other than those investigated in the fidelity assessment.
4. Lastly, no significant changes in unnecessary transfusions may be observed, despite good fidelity. If so, it could be inferred that the interventions are not effective and should not be scaled up into routine feedback practice. Alternatively, the process evaluation may identify unexpected factors that contributed to the observed changes in clinical practice across all participating hospitals, including contamination between trial arms, loss of treatment differentiation and contextual factors (i.e. new national transfusion guidelines).

Any such scenarios would provide valuable findings that would contribute to the scientific knowledge, evidence and theory regarding what makes for more effective A&F. Furthermore, an ongoing debate in the fidelity literature is that of ‘strict fidelity’ vs ‘adaptation’ (to key contextual factors), and the unresolved question of ‘how much’ fidelity is necessary or beneficial [12, 36]. For pragmatic trials such as the AFFINITIE cluster-RCTs, where variation and localised tailoring are likely inevitable, uniform or strict 100% fidelity may not be feasible to achieve nor necessarily desirable. General consensus in the literature is that 80 to 100% fidelity is considered ‘high’ and ‘good’ [12], yet there is insufficient evidence to support this. The proposed moderator/mediator analyses may identify that improved outcomes are achieved, for instance, with a ‘moderate’ degree of fidelity (e.g. 60%); thus implying a permissible degree of loss of intervention fidelity or adaptation. Such findings would be of interest to researchers developing future A&F interventions, as well as to policy makers and service commissioners, by providing feasibility data that may inform how and to what extent the interventions may be generalised in new settings or implemented on a larger scale, while maintaining similar benefits. This is the focus of Workstream 4 of the AFFINITIE programme (Fig. 1), which aims to explore lessons learned regarding A&F in the context of blood transfusion that may be generalised to A&F in other clinical contexts.

There are a number of limitations and challenges to the proposed process evaluation. The trials in AFFINITIE are pragmatic evaluations, embedded in the existing NHSBT NCA A&F programme. The trials and process evaluation are thus restricted by what is feasible to deliver and evaluate in this context. One notable challenge, highlighted in the intervention development process, is in describing ‘standard practice.’ A content analysis of existing NCA feedback reports and interviews with clinical transfusion staff highlighted significant variability in the content and structure of existing feedback reports and how hospitals currently respond to transfusion A&F. The lack of clarity as to what constitutes standard practice makes it difficult to establish at what point practice in the intervention arms differs from ‘usual practice,’ and whether or not practice has changed as a result of the interventions delivered.

An additional limitation, and potential source of contamination, is that members of the intervention development team are involved in conducting the process evaluation. This increases risk of biasing analysis of process evaluation data. The MRC process evaluation guidance [35] highlights the importance of being reflective of the relationship and boundaries between intervention developers, implementers and evaluators and recommends seeking occasional critical review from independent peers. The AFFINITIE Programme Steering Group provides oversight to the conduct of all elements of the programme and includes an independent chair and expert members who are familiar with the models and methods being used in AFFINITIE.

It has been argued that if process evaluations are to be funded and conducted, it is important that the protocol and findings from the process evaluation are transparently reported and published, as is standard practice for outcome evaluations [35, 37]. This is particularly important for fidelity investigations. While the BCC fidelity framework provides a synthesised conceptualisation and definition of different fidelity dimensions, the authors themselves acknowledge that there is little standardised guidance on how to operationalise each dimension, and a consequent need for further empirical examples of how each dimension has been quantified and interpreted [14, 38]. It is thus hoped that publishing the fidelity protocol and findings for the AFFINITIE process evaluation will provide a methodological contribution to the fidelity literature that may inform future fidelity assessments in other contexts.

## Conclusions

We have presented the protocol for a multidimensional assessment of intervention fidelity as part of the process evaluation of two enhanced A&F interventions to reduce unnecessary blood transfusions. It is anticipated that findings from the process evaluation will provide information critical to the interpretation of the trial outcomes. This protocol also contributes to the advancement of the process evaluation methods by illustrating the application of a comprehensive, multidimensional framework for assessing intervention fidelity, which may inform the design of future fidelity assessments.

## Additional file

Additional file 1: Sample semi-structured interview questions and questionnaire items for each domain in the Theoretical Domains Framework (DOCX 17 kb)

## Abbreviations

A&F: Audit and feedback
BCC: Behaviour Change Consortium
BCT: Behaviour Change Technique
NCA: National Comparative Audit
NHS: National Health Service
NHSBT: National Health Service Blood and Transplant
NICE: National Institute for Health and Care Excellence
RCT: Randomised controlled trial
TDF: Theoretical Domains Framework
