# Supplementary material for: A multidimensional approach to assessing intervention fidelity in a process evaluation of audit and feedback interventions to reduce unnecessary blood transfusions: a study protocol
Source: Implement Sci. 2016 Dec 12;11:163. doi: 10.1186/s13012-016-0528-x (PMC5153878; doi:10.1186/s13012-016-0528-x)
Supplement: Additional file 1: — Sample semi-structured interview questions and questionnaire items for each domain in the Theoretical Domains Framework (DOCX 17 kb) [file 13012_2016_528_MOESM1_ESM.docx]

**Supplementary Material 1*.*** *Sample semi-structured interview questions and questionnaire items for each domain in the Theoretical Domains Framework*

| **Domain** | **Sample interview question** | **Sample questionnaire item** |
| --- | --- | --- |
| **Knowledge** | Do you think the feedback materials showed that your site was doing well in relation to the PBM audit standards? | How well do you think the feedback materials showed that your site was doing compared with the audit standards?   \| **1** \| **2** \| **3** \| **4** \| **5** \| \| --- \| --- \| --- \| --- \| --- \| \| Not at all well \| Somewhat well \| No opinion \| Quite well \| Very well \| |
| **Skills** | Do you think your team/site has the necessary skills to change practice in light of feedback? | My team/site has the necessary skills to change practice in light of feedback   \| **1** \| **2** \| **3** \| **4** \| **5** \| \| --- \| --- \| --- \| --- \| --- \| \| Strongly disagree \| Disagree \| No opinion \| Agree \| Strongly Agree \| |
| **Social professional role and identity** | Just briefly, what is your role in the blood transfusion process?   - *Do you personally make decisions to transfuse?* - *How often do you make these decisions?* | Do you personally make decisions about whether or not to transfuse patients?   - Yes - No - Unsure/ Cannot remember   If yes, how often do you make decisions about whether or not to transfuse patients?   - Daily - Weekly - Monthly - Yearly - Occasionally - Unsure/ Cannot remember - Other (please specify) |
| **Beliefs about capabilities** | How confident are you in your ability to change your practice in light of feedback from the audit?   - *(If not applicable to their role)* *How confident are you that your team has the ability to change practice in light of feedback?* | I am confident in my ability to change practice in light of feedback   \| **1** \| **2** \| **3** \| **4** \| **5** \| \| --- \| --- \| --- \| --- \| --- \| \| Strongly disagree \| Disagree \| No opinion \| Agree \| Strongly Agree \| |
| **Beliefs about consequences** | What do you think are the downsides of changing blood transfusion practice in light of feedback?   - *What about the benefits?* | The feedback materials are likely to improve patient care   \| **1** \| **2** \| **3** \| **4** \| **5** \| \| --- \| --- \| --- \| --- \| --- \| \| Strongly disagree \| Disagree \| No opinion \| Agree \| Strongly Agree \| |
| **Motivation and goals** | Compared to other tasks that you have to do, where would you rank transfusion audit and feedback in terms of priority? | Compared to other tasks that you have to do, where would you rank transfusion audit and feedback in terms of priority?   \| **1** \| **2** \| **3** \| **4** \| **5** \| \| --- \| --- \| --- \| --- \| --- \| \| Lowest priority \| Quite low priority \| No opinion \| Quite high priority \| Highest priority \| |
| **Memory, attention and decision processes** | Do you remember which audit standards your hospital had particularly high achievement for?   - *What about standards for which your hospital had particularly low achievement for?* | Which of the following feedback materials from the audit do you recall receiving?   \| Feedback Material \| Received \| Not Received \| Unsure/ Cannot remember \| \| --- \| --- \| --- \| --- \| \| Key findings report \|  \|  \|  \| \| Full findings report \|  \|  \|  \| \| Regional slideshow \|  \|  \|  \| \| Toolkit \|  \|  \|  \| |
| **Environmental context and resources** | Are there any resource issues that affected your capacity to change transfusion practice in light of the audit findings? | My team/site has the necessary resources to change practice in light of feedback   \| **1** \| **2** \| **3** \| **4** \| **5** \| \| --- \| --- \| --- \| --- \| --- \| \| Strongly disagree \| Disagree \| No opinion \| Agree \| Strongly Agree \| |
| **Social influences** | Do other clinical staff/ colleagues influence your response to transfusion feedback?   - *How so?* - *How receptive have your clinical colleagues been to making changes in response to the audit findings?* | Other clinical staff/ colleagues influence my response to transfusion feedback   \| **1** \| **2** \| **3** \| **4** \| **5** \| \| --- \| --- \| --- \| --- \| --- \| \| Strongly disagree \| Disagree \| No opinion \| Agree \| Strongly Agree \| |
| **Emotion** | Did you enjoy reading the feedback reports? | The feedback reports were interesting to read   \| **1** \| **2** \| **3** \| **4** \| **5** \| \| --- \| --- \| --- \| --- \| --- \| \| Strongly disagree \| Disagree \| No opinion \| Agree \| Strongly Agree \| |
| **Behavioural regulation** | Did you make any plans on how to change your practice or procedures in light of feedback?   - *If so, can you talk me through how these plans were developed?* - *Did you use any of the feedback materials to develop your plans? If so, which?* - *Who was involved in developing these plans?* - *If not, can you talk me through potential reasons why you have not yet made any plans to change your practice or procedures?* | Do you keep monitoring your practice in light of feedback received and/or goals/plans developed for the PBM audit?  -Yes  - No  - Unsure/Cannot remember  If yes, please specify how you keep monitoring your practice (e.g. how often/by what means): |
| **Nature of the behaviour** | Has your response to this audit differed from what you normally do when receiving feedback from the National Comparative Audit of blood transfusion?   - *If yes, how so?* | To what extent has your response to the 2015 PBM audit differed from what you normally do when receiving feedback from the National Comparative Audit of blood transfusion?   \| **1** \| **2** \| **3** \| **4** \| **5** \| \| --- \| --- \| --- \| --- \| --- \| \| Not at all Different \| Slightly Different \| No opinion \| Quite a bit different \| Completely different \|     If slightly/quite a bit/ completely different, please tell us how your response has differed (e.g. new/additional activities you have undertaken as a result of the feedback): |
